# Maintenance of Pulp after Horizontal Root Fractures in Three Maxillary Incisors: A Thirteen-Year Evaluation 

**DOI:** 10.22037/iej.v12i4.16617

**Published:** 2017

**Authors:** Vânia Portela Ditzel Westphalen, Everdan Carneiro, Luiz Fernando Fariniuk, Ulisses Xavier da Silva Neto, Fernando Henrique Westphalen, Alexandre Kowalczuck

**Affiliations:** a * Department of Endodontics, School of Health and Biosciences, Pontifícia Universidade Católica do Paraná, Curitiba, Paraná, Brazil;*; b *Department of Oral Radiology, School of Health and Biosciences, Pontifícia Universidade Católica do Paraná and Department of Stomatology, School of Dentistry, Universidade Federal do Paraná, Curitiba, Paraná, Brazil *

**Keywords:** Connective Tissue Cells, Dental Pulp, Tooth Fractures

## Abstract

This case report documents the clinical approach adopted for three upper incisors with horizontal root fracture in the middle or cervical third. The proposed procedures involved maintaining pulp vitality and periodontal stability of the fractured teeth with 13 years of follow-up.

## Introduction

Root fractures mainly affect the maxillary central incisors, followed by the maxillary lateral incisors; the rate of occurrence of root fractures is 0.5 to 7% [1]. The most common fractures occur in the middle, followed by the cervical and apical third of the root [2].

A horizontal root fracture is diagnosed by clinical and radiographic examinations [3]. Clinical examinations include observation of the tooth position in the arch, assessment of the degree of mobility, presence of pain on impact and palpation, and response to pulp sensitivity tests. Radiographic analysis must be thorough and based on images acquired at different angles depending on fracture locations [3, 4].

Treatment consists of repositioning the crown fragment, stabilizing it with a fixed retainer followed by splinting to the adjacent teeth, and preserving pulp vitality and the type of repair around the fracture [3]. The desired condition for a tooth with a horizontal root fracture is the nearest possible approximation between the fragments with the deposition of mineralized tissue, as well as the maintenance of pulp vitality and slight mobility, similar to the physiological mobility of the intact tooth [3, 5].

This case report describes the occurrence of horizontal root fractures in the middle and cervical third of incisors that presented pulp vitality after 13 years of follow-up [6].

## Case Report

The patient was a 23-year-old woman who visited a dentoalveolar trauma clinic six months after a fall. The patient remembered being referred to a hospital and receiving initial care after the accident. An intraoral examination revealed the presence of rigid splint involving the maxillary incisors and canines. The left maxillary lateral incisor had a temporary crown 

**Figure 1:**
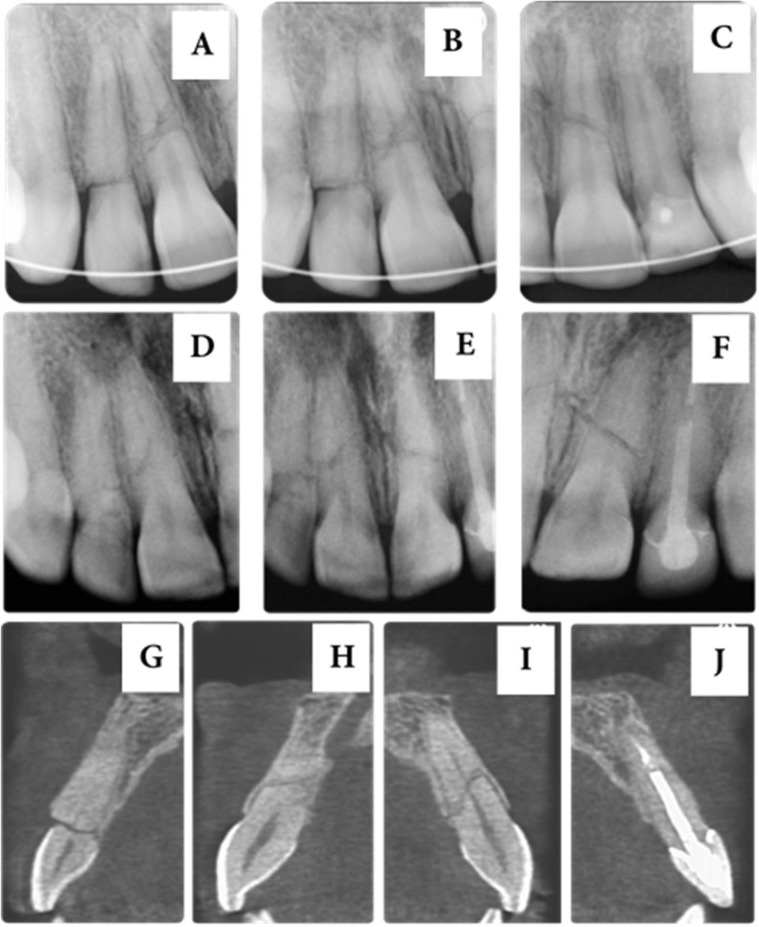
A to C) *Periapical radiographs acquired during initial care, demonstrating horizontal fractures in the cervical third of the right maxillary lateral incisor, and middle third of the right maxillary central incisors, and coronary fracture of the left maxillary lateral incisor*; D to F) *Periapical radiographs acquired 13 years after the initial treatment suggesting the formation of mineralized tissue between the fragments of the maxillary central incisors and the right maxillary lateral incisor*; G to J) *Computed tomographic images suggesting an aspect of normality for each fractured incisor*

made of composite resin without an intra-canal retainer; the other three maxillary incisors had crowns that were otherwise healthy. Radiographic examination revealed complete root formation in the involved teeth, a root fracture in the cervical third of the right maxillary lateral incisor (Figure 1A), and a fracture in the middle third of the maxillary central incisors (Figure 1B and 1C). All teeth had positive response to pulp sensitivity tests.

During a second visit, the patient underwent endodontic treatment in the right maxillary lateral incisor to allow for the installation of a root canal seal and for prosthetic rehabilitation of the crown. During the first six months, monthly radiographic and clinical controls of all the involved teeth were performed. Dental splint was maintained for 12 months because of the right maxillary lateral incisor fracture. 

At the time of splint removal, the teeth did not show increased mobility. The maxillary central incisors and right maxillary lateral incisor showed positive responses to pulp sensitivity tests. Annual controls were performed until 2009. Because the patient did not attend the scheduled controls in the following years, the subsequent follow-up was possible only in February 2016. Clinical examination revealed normal soft and hard tissues, and a positive response to pulp sensitivity tests. Periapical radiographs (Figure 1D to 1F) and a computed tomography (CT) examination were conducted for evaluating the involved teeth.

The tomographic image suggested repairing of the maxillary central incisors with deposition of mineralized tissue between the fragments (Figure 1G to 1J). The right maxillary central incisor appeared to have multiple fractures with a slight reduction in the root canal volume, especially in the apical fragment, in addition to rounding of the fragments.

## Discussion

The distance between the fragments and the level of horizontal root fracture directly interfere with the treatment outcome. The determination of these parameters involves interpretation of the radiographic image, which allows classification of the fractures as follows: cervical, middle, or apical [2].

The adoption of CT as a diagnostic aid adds much greater complexity to fracture classification. The comparison between radiographic and tomographic images of teeth with root fractures showed no agreement in the level of fractures[7]. In the present case, the radiographic image of the right maxillary central incisor hints, at certain angles, at an oblique fracture (Figure 1B to 1E). The tomographic image reveals the separation of the root into several fragments, not just into the coronary and apical. CT is particularly important in cases of multiple fractures in the same root [8], and in the alteration of the long axis of one of the fragments [9]. However, in this case, even though the patient had several fractured teeth and complex fractures, a CBCT scan was not available at the time of initial care. Therefore, radiographs at different angles were acquired for evaluation. Currently, the use of CBCT during initial care would be clearly justified in such a case. The involvement of four teeth in an accident makes a large number of radiographic examinations (occlusal and periapical) necessary in order to understand the configuration of each fragment involved. After radiographic confirmation of the fracture, CBCT can be suggest in order to clarify the extent and direction of fracture, because two-dimensional limitation of radiographic view [7].

Pulp vitality is better preserved in teeth that have undergone horizontal fractures than in teeth with dislocations and without root fractures[10]. The rapid reduction and immobilization of the fracture was instrumental in maintaining pulp vitality of the involved teeth. Although the fracture of the right maxillary lateral incisor occurred in the cervical third, maintaining pulp vitality is proof of the absence of contamination of the root canal. The exposure of the fracture to contamination of the gingival sulcus makes it more difficult to stabilize the fragment in the cervical portion and compromises tissue repair in the cervical third [11, 12]. Only 30% of fractured teeth in the cervical third present pulp vitality. The maintenance of splint for more than the recommended time had a positive influence on the stabilization of this tooth. After 12 months, it was possible to observe a lack of mobility in the coronary fragment of the right maxillary lateral incisor [3, 13]. Andreasen *et al*. (13) evaluated healing process between fragments when root fracture was verified. Although no difference was observed for healing of teeth splinted for two months or less and for longer splinting periods. A particular situation could happen to cervical fractures, when longer time of maintenance of splint could be necessary. In present case teeth had a positive response to pulp sensitivity tests, but increased mobility was detected during the first six months of follow-up. For this reason longer time of splint was proceeded.

The prognosis for middle third fractures is more favorable, and in about 86% of cases, it is possible to maintain the responsiveness of the affected teeth to pulp sensitivity tests [14]. The diameter of the apical foramen, condition of vascular support, number of cells available and degree of diastasis (separation between fragments) are fundamental to the prognosis [15]. When there is little movement between the fragments, it is possible to have tissue repair without incidentally discovered during radiographic examinations requested for other purposes [8, 16, 17]. 

The recommended duration of follow-up for cases of root fractures is currently at least five years [3]. The reported clinical case was originally published in 2008, after five years of observation [6], and clinical control and CT examination were performed after eight years. 

## Conclusion

Although the present case suggests 13 years of stability, clinical and radiographic success, routine follow-ups are still required. Periodontal problems resulting from poor hygiene can trigger cervical resorption in teeth with root fractures similar to those observed. 
